# ECG Abnormalities in Patients with Acute Exacerbation of Bronchiectasis and Factors Associated with High Probability of Abnormality

**DOI:** 10.1155/2021/6649572

**Published:** 2021-07-05

**Authors:** Fatima Alhamed Alduihi

**Affiliations:** Department of Internal Medicine, Aleppo University, Aleppo, Syria

## Abstract

**Background:**

Bronchiectasis is an important reason for morbidity and mortality according to the last records that referred to high incidence rate of disease. Cardiovascular problems are common in pulmonary diseases, in general, and it can symptom by ECG abnormalities. The objective of this study was to define the most ECG abnormalities in patients with acute exacerbation of bronchiectasis and to study the correlation between the cardiac disorder and the other risk factors of the exacerbation.

**Materials and Methods:**

A prospective single-center observational cohort study was done at Aleppo University Hospital for patients with AEB between October 2017 and September 2018. They were divided into 2 groups (normal ECG vs. abnormal). Patients with COPD, cystic fibrosis, new diagnosis of ischemic accident through the last 6 months of the study, and treatment with macrolides or fluoroquinolones through the last 3 months of the study were excluded. We study the percent of abnormalities through the AEB and the percentage of the most common abnormalities.

**Results:**

67 patients were included in the study (44 males and 23 females) with a mean age of 52.85 ± 21.456. ECG abnormalities were recorded in 43 patients, and it was more common in men (67.44% of cases). Advanced age and survival state had a statistical significance (*p* = 0.003, 0.023), respectively, between the 2 groups. Right axis deviation (RAD) is the most common abnormality (23.3%) followed by sinus tachycardia (20.9%), and it is close to T-depression (18.6%). AF was the most common arrhythmia from all recorded arrhythmias (6.98% from all cases). Positive sputum cultures were recorded in 55.8%, and the most common isolated pathogen factor was *Pseudomonas aeruginosa*. Recurrent pneumonia was seen in 30.2% of all patients with abnormal ECG. We find a high prevalence of ECG abnormalities in patients with Oximetry (90-95%, 39.5%), and the opportunity for abnormalities is equal in the 2 age groups (45–59 and more than 75) that reflexed the possibility of cardiac disorders in any age in patients with AEB.

**Conclusions:**

ECG abnormalities are common in AEB, and it can happen in any age and any value of Oximetry. It needs more attention because of the prognosis of the cardiac morbidity.

## 1. Introduction

Bronchiectasis is one of the most important reasons for attending the respiratory clinics.

Recent recodes refer to higher rates of prevalence in patients from all ages, and there is a notable peak on young ages [1–4].

The heart is the organ close to the lungs from anatomic side, so it will be affected by lesions that affected the lungs like COPD and bronchiectasis. This study is aimed at evaluating the function of the heart through bronchiectasis by performing a simple procedure which is usually applied to all patients admitted to hospital (electrocardiography (ECG)).

## 2. Patients and Methods

### 2.1. Patient Population

A prospective retrospective observational one-center study was applied to patients who were admitted with the diagnosis of acute exacerbation of bronchiectasis (AEB) in the period between October 2017 and September 2018 in Aleppo University Hospital, Aleppo, Syria.

They were divided into 2 groups (normal ECG and abnormal). Patients with COPD, cystic fibrosis, long treatment or treatment through the last 3 months with macrolide or fluoroquinolones, previous diagnosis of arrhythmia, and new ischemic accident (brain or cardiac infarction) through the last 6 months were excluded.

### 2.2. Data Collection

We reviewed and followed the patients' profiles and collected the following variables: demographics, smoking history, diagnosis of COPD or CF by a physician, sputum (and/or) tracheal cultures on admission, oxygen saturation on admission, medications, and morbidity.

The study is approved by the local ethical committee, and every patient agreed to continue participation in our study. Each patient gave his or her written consent for the collection and use of their clinical data.

### 2.3. Statistical Analysis

Demographic characteristics and variables of interest were summarized by the state of ECG (normal ECG vs. abnormal ECG) using descriptive statistics: mean (SD) for continuous variables and frequency (proportion) for categorical variables.

To assess the association between demographic characteristics and risk, logistic regression was used, and the results are reported as an odds ratio, with a 95% confidence interval.

A *p* value of less than 0.05 was used to detect the statistical significance. *χ*^2^ is used as a test for statistical significance. Analysis was performed using IBM SPSS statistical software, V24.

## 3. Abbreviations Used in the Manuscript

The following abbreviations are used: ECG (electrocardiography), COPD (chronic obstructive pulmonary disease), CF (cystic fibrosis), RAD (right axis deviation), LAD (left axis deviation), LVH (left ventricle hypertrophy), HTN (hypertension), MAT (multifocal atrial tachycardia), PVC (premature ventricular complex), AEB (acute exacerbation of bronchiectasis), RBBB (right bundle-branch block), LBBB (left bundle-branch block), AF (atrial fibrillation), and AAA (acute asthma attack).

## 4. Results

67 patients were included in the study (44 males and 23 females) with a mean age of 52.85 ± 21.456 years old.

ECG abnormalities were recorded in 43 patients (64.17% of patients), and they were more common in men (29, 67.44% of all 43 cases).

Abnormal ECGs were recorded in patients with advanced age more than in younger ages. In a previous ischemic accident, cerebral or cardiac disorder was recorded in 20 cases (29.85%); 17 of them recorded abnormal ECGs (39.5%). Six of them have ages less than 60 years and 14 have ages more than 60 years.

Mean Oximetry (oxygen saturation) was 87.81 ± 10.123%, and it had higher values in patients with normal ECG.

40 patients were smokers (59.7%): 12 of them have normal ECG (30% of all smokers), and 28 of them have abnormal ECG (70%).

13 patients died through one year of follow-up; only one of them has normal ECG, while 12 have abnormal ECG. The mean age of dead patients was 68.15 years: the oldest one was 99 years old, and the youngest was 15 years old.

Positive sputum cultures were recorded in 42 patients (62.68% of all cases); 24 of them (55.8%) had abnormal ECG, and 8 (33.33%) of them showed *Pseudomonas aeruginosa* in the result of culture, the most isolated pathogen from all the positive cultures.

Recurrent pneumonia was the most common trigger factor of exacerbation in 23 patients (34.32%). 30.2% of the patients with abnormal ECG had recurrent pneumonia.

Pleural effusions were seen only in patients with abnormal ECGs (4 patients) and also autoimmune diseases (5 cases, 4 abnormal, and 1 normal).

Bronchitis was investigated in 7 patients (10.44%); 5 of them had normal ECG, while 2 had abnormal ECG.

Increased dyspnea was a stable variant in 65 patients (97% of all patients in the study); it was seen in all patients with normal ECG and in 41 patients with abnormal ECG.

Demographic characteristics and both the likelihood ratio and *p* value were calculated and are summarized in Table 1.

Right axis deviation (RAD) was the most common abnormality in 23.3% of cases followed by sinus tachycardia (20.9%), and it is close to T-depression (18.6%).

Both the left axis deviation (LAD) and left ventricle hypertrophy (LVH) recorded the same percent (4.7%) which reflexes the morbidity of hypertension (HTN) in such patients.

Less frequent abnormality was recorded equally for multifocal atrial tachycardia (MAT) and premature ventricular complex (PVCs) (2% for every abnormality).

**Figure 1:**
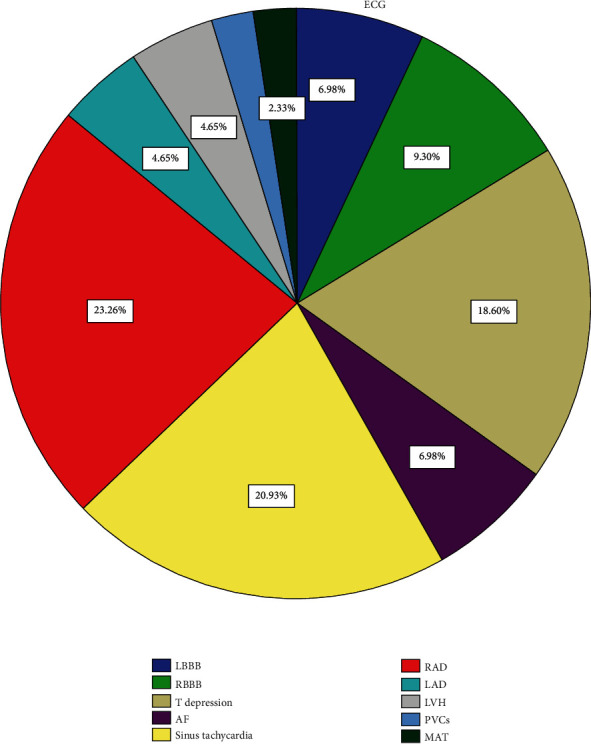
The most common ECG abnormalities in patients included in our study with their percent of each abnormality.

Figure 1 shows the most common ECG abnormalities in patients who were included in our study with their percent of each abnormality.

What was really abnormal was a high prevalence of ECG abnormalities which was recorded in patients with oxygen saturation (90-95%, 39.5%), and the opportunity for abnormalities is equal in the 2 age groups (45–59 and more than 75), which reflexed the possibility of cardiac disorders in any age and in any value of saturation in patients with acute exacerbation of bronchiectasis (AEB).

Mortality, confirmed previous ischemic accident, and age had a statistical significance (*p* = 0.023, 0.025, and 0.003), respectively.

**Figure 2:**
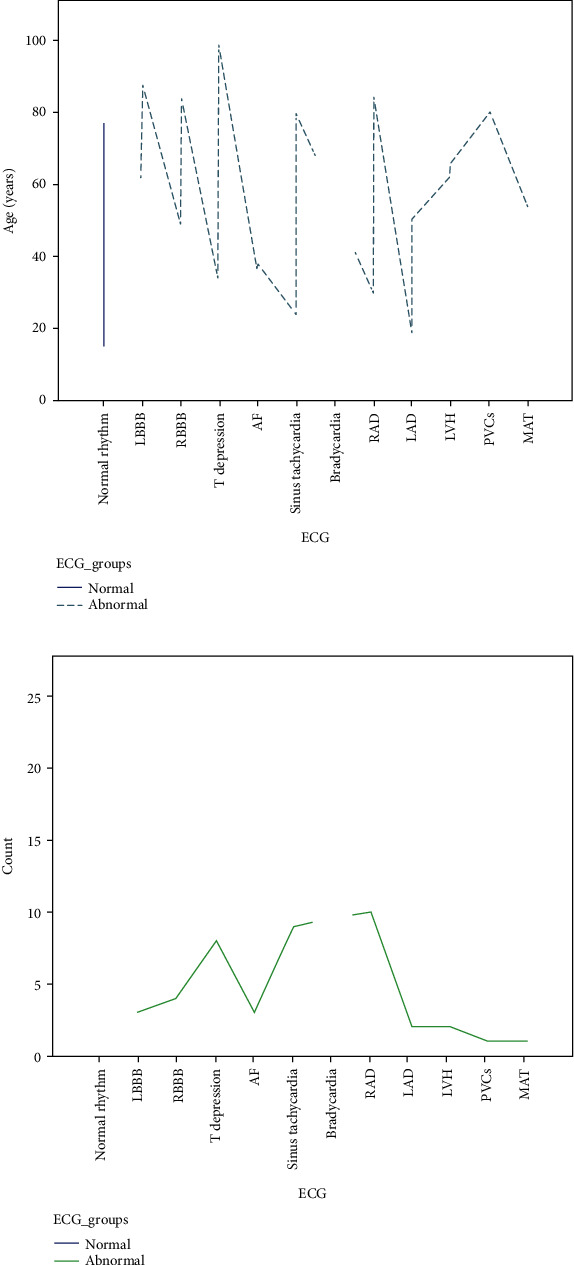
ECG records of the patients of study. (a) ECG changes according to the age. (b) ECG changes in all cases. MAT: multifocal atrial tachycardia; PVCs: premature ventricular contractions; LVH: left ventricle hypertrophy; LAD: left axis deviation; RAD: right axis deviation; AF: atrial fibrillation; RBBB: right bundle branch block; LBBB: left bundle branch block.

Figure 2 shows the graphs of the most common abnormalities according to the age.

## 5. Studying the Risk Estimate

To assess the association between demographic characteristics and risk of developing abnormal ECG, logistic regression was used, and the results were reported as an odds ratio, with a 95% confidence interval.

We divided patients according to age (less than 60 years and more than 60 to be more accurate regarding the age, and we did not find an important statistical significance). We also studied the oxygen saturation between 2 values (less than 92% and more than 92%).

The odds ratios for the variants are printed on Table 2.

## 6. Discussion

The study highlighted the fact of the correlation between bronchiectasis and ECG abnormalities, as they were found in most of the patients with a high rate of 64.17%.

Cardiac disorders are common in respiratory disease in general and especially in chronic lung diseases [5]. There were many records about the increased risk of cardiovascular diseases in patients with bronchiectasis [6, 7].

The cardiac comorbidity can be a result of the disease itself because of anatomical correlation between the lung and the heart (as in the pulmonary hypertension for example) or because of the treatment of pulmonary diseases that may affect the heart; macrolides are used widely in the treatment of community-acquired pneumonia and bronchiectasis especially in cases of long-term treatment [8–10]. The fluoroquinolone group, another drug group used widely in the treatment of bacterial exacerbations of obstructive diseases, can increase the risk of arrhythmias [11–13].

ECG is a simple procedure. It can direct the physician or clinician to misdiagnosed cardiac comorbidity in patients with bronchiectasis or any other obstructive lung disease and hypertension in patients with marks of LVH on the ECG for example.

Male gender is a risk factor for developing abnormal ECG compared with women; however, there is no important statistical significance (*p* = 0.79) that refers the incidence of abnormalities as close between patients [14, 15]. Abnormal ECGs were more recorded in smokers, accepted in many studies in this field that confirmed the correlation between abnormalities and smoking [16, 17]. Mortality was higher between patients with abnormal ECGs, whose ages were advanced compared with those of young patients. This result confirmed the fact of the poor prognosis of patients with cardiac comorbidity compared with those who do not have this problem, especially in advanced ages. Age itself can be a risk factor for mortality in patients with AEB [1, 18, 19]. The likelihood ratio recorded the highest rate for age in our study (45.46). It suggested age as a reason for abnormalities by itself. It added another risk and enhanced the morbidity of the disease. Both cardiac morbidity and advanced age enhanced together the risk of mortality in such group of patients [20–22].

Oximetry (oxygen saturation) had high values in patients with normal ECG compared with those with abnormal ones, accepted in Kellett et al. [23] and Katz et al. [24], that suggested abnormalities in ECG as a result of hypoxemia in bronchoscopy, and it was so close to Romero and Jané's study [25] that suggested hypoxia as a reason for abnormalities in ECG. In a review, there were many studies suggesting hypoxia as a reason for ECG abnormalities in patients with obstructive pulmonary diseases [26, 27].

Dyspnea was a constant symptom in all patients, and there is no statistical significance between the 2 groups of patients even though it had more risk to develop abnormalities on ECG (odds ratio, 1.585). Increased dyspnea had a likelihood of 1.808 for developing abnormal ECGs. Dyspnea can cause abnormalities in cases that lead to a metabolic acidosis [28]. Dyspnea induced by exercise in children and adolescents usually made a tachycardia, sinus, or ventricle [29, 30]. Our study showed that dyspnea was found in all patients with no differences between the 2 groups of study, normal and abnormal ECG. Nine patients with dyspnea had sinus tachycardia as the most common abnormality in the group of abnormal and also RAD.

Recurrent pneumonia triggers the exacerbation of bronchiectasis in 23 patients (34.32% of all cases, 30% of the abnormal ECG group), which confirms the etiology (postinfections) as a most common etiology in bronchiectasis [31].

Positive isolated cultures were more common in the group of abnormal ECGs, but there are no differences between the 2 groups. *Pseudomonas aeruginosa* was the most common isolated pathogens referred by most studies all over the world [32–35].

RAD is the most common abnormality (10 patients, 23.3%); 9 of them 90% had dyspnea.

Right bundle-branch block (RBBB) had been recorded in 9.30% (4 patients). These abnormalities correlated mainly with disorders in the function of the right ventricle that carried a specific importance to have a right catheterization for pulmonary hypertension diagnosis after echocardiography was not enough in such patients [36, 37]. Another specialty of RAD is the correlation with the cases of PCD, as it is the most ECG abnormality in patients with Kartagener disease [38–40]. Three of the patients included in the study had the diagnosis of Kartagener disease, and some had a dextrocardia and RAD on ECG.

Sinus tachycardia was recorded in 9 cases (20.9%), all of them had dyspnea, and it was very close to T-depression (8, 18.6%) which reflexed the role of infectious state on the ECG, as tachycardia is a result of infection and high temperature [41]. It carries a bad predictor of poor prognosis especially in patients with severe asthma in cases that the exacerbation of asthma was a trigger factor of the exacerbation of bronchiectasis [42]. As asthma was recorded in 6 patients (8.95% of all cases in the study), it was important to suspect a severe infection and a poor prognosis outcomes in patients with sinus tachycardia as postinfection (recurrent pneumonia), and exacerbations of asthma had a high prevalence between the patients of the study.

T wave depression is a sign of wide spectrum of cardiac and noncardiac disease [43, 44]. It can be a sign of ischemic accident (ischemic heart disease [45], pulmonary edema [46], and heart failure in athletes [47]). T wave depression had its importance because of the underlying ischemic etiology, and in our study, 6 patients of the 8 patients had a previous ischemic accident. That really confirmed the comorbidity of ischemic accidents and patients with acute exacerbation of bronchiectasis.

Hypoxia is an important reason for inversion of T wave [48]. Patients with acute respiratory alkalosis present more danger than those with chronic because they are more likely to develop metabolic compensation [49], and as a final result, a case of uncompensated acidosis can cause inversion in T wave [50, 51].

Both the left bundle-branch block (LBBB) and atrial fibrillation (AF) have a 6.98 percentage for each one. LBBB was suspected to be a risk factor for cardiovascular morbidity in patients with chronic obstructive pulmonary diseases [52], so that will be a risk factor for cardiac disorders in patients with overlap syndrome (bronchiectasis-COPD).

Atrial fibrillation was the most common recorded arrhythmia in patients with chronic pulmonary diseases, especially those associated with pulmonary hypertension [53] and COPD [54]. It was one of the important findings of the study even with excluded patients with COPD; AF is still on the top of arrhythmias in patients with acute exacerbation of bronchiectasis. Other arrhythmias were less frequent like MAT and PVCs (2.3% for each one).

A study of arrhythmias in all the included patients shows that they were recorded in only 5 cases (11.62% from all abnormalities), so it was not common in our study compared with other ECG abnormalities.

Both the left axis deviation (LAD) and left ventricle hypertrophy (LVH) had a 4.65 percentage, which is correlated with an increased high risk of hypertension comorbidity in these patients [55].

As COPD correlated with a lot of cardiac disorders [56–58] and was suspected as a risk factor for cardiovascular comorbidity in patients with emphysema [59, 60] and at last was recorded to have a high rate of arrhythmias [61, 62], it was important to exclude the patients with COPD from the study.

## 7. Limitations of the Study

There are a small number of patients compared with international studies, as there is no registry for bronchiectasis in Syria.

It was difficult to exclude all patients with previous ischemic accidents because of the fact that there is a high prevalence in elderly patients and those who had much comorbidity in our region.

We did not assess the severity of AEB according to faced or BSI scores because not all patients accepted had lung function diseases, and we could not depend only to the previous records; the severity of disease was assessed by advanced lesions on HRCT.

## 8. Strong Points

This was the first study to suggest ECG abnormalities as a predictor of cardiovascular morbidity in patients with acute exacerbation of bronchiectasis. Patients of COPD were excluded to be more accurate and definite to bronchiectasis without overlap syndrome with COPD.

## 9. Conclusions

ECG abnormalities are common in patients with acute exacerbation of bronchiectasis. It can happen in any age and any value of oxygen saturation (Oximetry). Advanced age and survival state had a statistical significance (*p* = 0.003 and 0.023), respectively, between the 2 groups.

Male gender, smoking, and increasing dyspnea are factors with more likely opportunity for developing abnormalities on ECG.

## Figures and Tables

**Table 1:** Demographic characteristics for the patients of the study.

	All (*N*: 67)	Normal ECG (*N*: 24)	Abnormal ECG (*N*: 43)	Likelihood ratio	*p* value
Gender					
Male	44 (65.67%)	15 (62.5%)	29 (67.44%)	0.166	0.79
Female	23 (34.33%)	9 (37.5%)	14 (32.65%)		
Smoking	40 (59.70%)	12 (50%)	28 (65.11%)	1.454	0.3
Mortality (1 year)	13 (19.69%)	1 (4.16%)	12 (27.90%)	6.698	0.023
Previous ischemic accident	20 (29.85%)	3 (12.5%)	17 (39.53%)	5.888	0.025
Increased dyspnea	65 (62.68%)	24 (100%)	41 (95.34%)	1.808	0.533
Age (years)	52.85 ± 21.456.	43.33 ± 20.599	59.3 ± 19.939	45.46	0.003
Positive cultures	42 (62.68%)	18 (75%)	24 (55.81%)	2.501	0.187
Oximetry (%)	87.81 ± 10.123	90.38 ± 7.347	86.09 ± 11.196	8.758	0.227
Trigger factors					
AAA	6 (8.95%)	2 (8.33%)	4 (9.30%)	15.656	0.113
Recurrent pneumonia	23 (34.32%)	10 (41.66%)	13 (30.23%)		
Pleural effusion	4 (5.97%)	0	4 (9.30%)		
Autoimmune	5 (7.46%)	1 (4.16%)	4 (9.30%)		
Abscess	2 (2.98%)	1 (4.16%)	1 (2.32%)		
Exposure	6 (8.95%)	4 (16.66%)	2 (4.65%)		
Bronchitis	7 (10.44%)	5 (20.83%)	2 (4.65%)		
Another	14 (20.89%)	3 (12.5%)	11 (25.58%)		

*p* value of less than 0.05 was used to detect the statistical significance, and *χ*^2^ is used as a test for statistical significance.

**Table 2:** Risk estimate in the study.

Variant	*p* value	Odd ratio	Confidence interval (95%)	
			Low	Upper
Gender (man/women)	0.79	1.083	0.733	1.600
Smoking (smoking/nonsmoking)	0.3	1.260	0.850	1.868
Mortality (nonsurvivor/survivor)	0.023	1.329 0.149	1.084 0.021	1.630 1.097
Previous ischemic accident (yes/no)	0.025	1.564	1.133	2.159
Dyspnea (increased)	0.533	1.585	1.316	1.909
Age (less than 60/more than 60)	0.072	0.707	0.501	0.999
Oximetry (less than 92/more than 92)	0.109	1.422	0.900	2.247
Positive cultures (yes/no)	0.187	0.752	0.534	1.059

*p* value of less than 0.05 was used to detect the statistical significance, and *χ*^2^ is used as a test for statistical significance.

## Data Availability

The data are available from the corresponding author on a reasonable request.
